# Evaluation of the relationship between diabetic retinopathy and left atrial deformation parameters

**DOI:** 10.1186/s43044-022-00265-x

**Published:** 2022-04-13

**Authors:** Şeyhmus Külahçıoğlu, Işıl Kutlutürk Karagöz, Yusuf Bilen, Barkın Kültürsay, Ravza Betül Akbaş, Enver Yücel, Hacer Ceren Tokgöz, Abdülkadir Uslu, Ali Karagöz, Cihangir Kaymaz

**Affiliations:** 1 Department of Cardiology, Kartal Koşuyolu Education and Research Hospital, Kartal Koşuyolu Yüksek İhtisas Eğitim ve Araştırma Hastanesi, Denizer caddesi Cevizli Kavşağı No: 2, Kartal, stanbul, Turkey; 2 Department of Ophtalmology, Şişli Hamidiye Etfal Training and Research Hospital, Istanbul, Turkey; 3 Department of Cardiology, Sancaktepe Training and Research Hospital, Istanbul, Turkey

**Keywords:** Speckle tracking echocardiography, Left atrial contractile strain, Diabetic retinopathy

## Abstract

**Background:**

Left ventricular systolic dysfunction (LVSD) may develop without coronary artery disease, hypertension (HT), or valvular pathologies in patients with diabetes mellitus (DM), which is defined as diabetic cardiomyopathy (DCM) and its pathophysiology is still unclear. Diabetic retinopathy (DR) is a microvascular complication of DM, and patients with DR have increased risk for the development of heart failure (HF). Two-dimensional speckle tracking echocardiography (2D-STE) evaluates longitudinal deformation in left atrium (LA) myocardium and previous studies utilizing 2D-STE have revealed the detrimental effects of DM on LA functions. Although some studies have shown the association between DR and left ventricle (LV) systolic functions, as far as the researchers of this study investigated, there is no study evaluating the relationship between LA deformation parameters and DR. Hence, we aimed to investigate the relationship between the presence and the degree of DR and LA deformation parameters.

**Results:**

LA deformation parameters were analyzed in terms of LA reservoir, conduit, and contractile functions according to the degree of DR. LA reservoir strain value was 14.2 ± 3.6 in normal retina group, 12.2 ± 4.1 in non-proliferative diabetic retinopathy (NPDR) group, and 13 ± 3.7 in proliferative diabetic retinopathy (PDR) group (*P* = 0.04). LA contractile strain was 15.9 ± 6.8 in normal retina group, 13.1 ± 47.4 in NPDR group, and 9.9 ± 4.7 in PDR group (*P* < 0.001). LA conduit strain was 30.1 ± 6.6 in normal retina group, 25.3 ± 6.5 in NPDR group, and 22.9 ± 4.9 in PDR group (*P* < 0.001). Proportional odds regression for association between clinical data, echocardiographic parameters, and LA contractile strain function showed that increasing creatinine (from 0.7 to 1.0; OR 0.71; 95% CI 0.51–0.99; *P* = 0.04), DR presence (OR 0.24; 95% CI 0.11–0.50; *P* = 0.001), and increasing left atrial volume index (LAVI) (from 33.5 to 52.6; OR 0.62; 95% CI 0.43–0.89; *P* = 0.01) were associated with decreasing LA function; however, other variables indicated no association.

**Conclusions:**

Our results showed the relationship between LA deformation parameters and DR, although microvascular involvement is not a certainly defined cardiovascular risk factor. Further prospective studies are needed to determine the clinical importance of DR presence and its degree for deformation parameters.

## Background

Diabetes mellitus (DM) and the related cardiovascular complications are the important causes of morbidity and mortality worldwide. In patients with DM, left ventricular systolic dysfunction (LVSD) may develop without coronary artery disease, hypertension (HT), or valvular pathologies, and which is defined as diabetic cardiomyopathy (DCM). Although the pathophysiology of DCM is still unclear, oxidative stress, renin-angiotensin system activation, impaired mitochondrial function, microangiopathy, hyperinsulinemia, insulin resistance, hyperglycemia, and hyperlipidemia have been suggested as most possible contributors [1, 2].

Diabetic retinopathy (DR) is a specific and common microvascular complication of DM. Studies have shown that the presence of DR has a high risk for heart failure (HF), independent of well-known cardiovascular risk factors [3].

In DCM, diastolic dysfunction (subclinical in general) is the most common cardiac manifestation thought to occur secondary to microvascular disease, and its association with DR supports this etiology [4]. In patients with or without microvascular complications, the presence of subclinical LVSD -even before diastolic dysfunction- could not be detected with routine transthoracic echocardiographic (TTE) assessment. Hence, for early diagnosis, the two-dimensional speckle tracking echocardiography (2D-STE) gains importance in patients with DM and preserved left ventricle ejection fraction (LVEF) [5].

The left atrium (LA) has multiple functions like contraction, conduit, and reservoir. LA interacts with the LV, the latter chamber fills. The prognostic role of the LA function in several conditions such as atrial fibrillation, HF, stroke, and valvular heart diseases has been described in previous studies [6].

2D-STE evaluates longitudinal deformation in the LA myocardium and previous research utilizing 2D-STE have revealed the detrimental effects of DM on the LA functions [7, 8]. Although studies have shown the association between DR and LV systolic functions [9], as far as the researchers of this study investigated, there is no study evaluating the relationship between LA deformation parameters and DR. Hence, in this study, we aimed to investigate the relationship between the presence and the degree of DR and LA deformation parameters using 2D-STE technique.

## Methods

In this single-center retrospective observational study, patients with DM, and who had been evaluated for retinopathy and referred to our outpatient cardiology clinic for cardiovascular system examination between September 2020 and April 2021 were included. The medical history, electrocardiogram and other noninvasive/invasive test results, physical examination, laboratory findings, echocardiography reports, as well as the results of LA strain analysis and 2D and Doppler echocardiography images were obtained from the hospital electronic database.

Patients with known coronary artery disease, those with ischemia diagnosed by noninvasive methods, and subjects with moderate to severe mitral valve regurgitation and without LA strain analysis were excluded from the study. Finally, a total of 147 patients met the inclusion criteria and were enrolled.

GE Vivid 7 ultrasound system was utilized for echocardiographic evaluation of the patients. Chamber diameters, wall thicknesses, LA volumes, and LVEF were assessed according to the recommendations of the European Association of Cardiovascular Imaging (EACI). Moreover, LA strain analysis was performed using 2D-STE with Echo-Pac software package designed for LV strain evaluation, according to the previous guidelines [8, 10] and LA strain was also evaluated with this software.

The local ethical committee approved the study and all patients signed a written informed consent.

### Statistical analysis

Numerical data were presented as mean and standard deviation (SD). Categorical data were defined as frequency and percentage. We used Student’s t-test and analysis of variance (ANOVA) to compare continuous data, and Pearson’s Chi-Square test was used for comparison of categoric data. To determine independent predictors for dependent variable (LA strain parameters function), crude and adjusted proportional odds regression analyses were used.

#### Outcome variable

Left atrial strain parameters (contractile strain).

#### Statistical modeling

Multivariable proportional odds regression models were used. Predictors (confounders) of multivariable were selected according to previous literature. *P*-value less than 0.05 was defined as a statistical significance. Statistical analyses were performed using R 4.04 software (Vienna, Austria).

## Results

In this study, a total of 147 diabetic patients were enrolled. The baseline characteristics of the patients according to the presence or absence of retinopathy are presented in Table 1. All patients were diagnosed with DM. The mean value of HbA1c (%) in the normal retina group was 7.3 ± 0.9, while it was 8.9 ± 1.9 in the retinopathy group. The mean age in patients without retinopathy was 52.6 ± 9.3, while it was 55.4 ± 10.3 in the retinopathy group. HT was present in 33 (66.7%) and 68 (58.6%) patients in normal retina and retinopathy groups, respectively. Other clinical and laboratory parameters are shown in Table 1.

**Table 1:** Comparison of baseline clinical and laboratory parameters in diabetics with normal retina versus retinopathy

Variable	Normal Retina, (*n* = 51)	Retinopathy present, (*n* = 96)	*P*-value
Age, (years)	52.6 (9.3)	55.4 (10.3)	0.09
Gender (Female), *n* (%)	36 (66.7)	62 (53.4)	0.14
HT, *n* (%)	36 (66.7)	68 (58.6)	0,40
Systolic BP (mm Hg)	135.3 (15.8)	137.6 (19.4)	0.44
Diastolic BP (mm Hg)	79.1 (10.4)	82.9 (12.6)	0.06
Smoking, *n* (%)	8 (14.8)	42 (36.2)	0.008
Lipid lowering therapy, *n* (%)	22 (40.7)	18 (15.5)	0.001
Glucose (mg/dL)	161 (53.7)	235 (93.5)	< 0.001
Creatinine (mg/dL)	0.8 (0.3)	0.9 (0.3)	0.03
Albumin (mg/dL)	4.3 (0.4)	4.5 (0.4)	0.04
LDL (mg/dL)	91.5 (23.7)	121.4 (46.3)	0.004
TSH (mIU/L)	1.5 (0.7)	1.9 (0.5)	0.52
Microalbumin/creatinine (spot urine)	7.60 (3.60)	19.4 (10.5)	< 0.001
WBC (10^3^/µL)	7.1 (1.6)	7.7 (1.7)	0.46
Hemoglobin (g/dL)	12.5 (1.5)	13.1 (1.4)	0.005
HbA1c (%)	7.30 (0.9)	8.9(1.9)	< 0.001
hs-CRP (mg/L)	0.6 (0.7)	0.6 (0.8)	0.74

Continuous variables are presented as mean and standard deviation (SD)

BP, Blood pressure; LDL, low-density lipoprotein; TSH, Thyroid stimulating hormone; WBC, White blood cell; hs-CRP, High sensitive C-reactive protein

LVEF, end-diastolic volume, left atrial volume index (LAVI), and LV-twist values were similar between normal retina and retinopathy groups; however, mitral annular plane systolic excursion (MAPSE) decreased in retinopathy group (13 ± 2 and 12 ± 2, respectively; *P* = 0.004). Also, global longitudinal strain (GLS) was lower in retinopathy group compared to normal retina group (− 18.9 ± 2.1 and − 17.8 ± 2.7, respectively; *P* = 0.008). Other echocardiographic parameters are presented in Table 2.

**Table 2:** Comparison of basic echocardiographic parameters in diabetics with normal retina vs. retinopathy

Variables	Normal retina, (*n* = 51)	Retinopathy present, (*n* = 96)	*P*-value
LV septal wall (cm)	1.1 (0.1)	1.1 (0.1)	0.11
LV posterior wall (cm)	1 (0.1)	1.1 (0.1)	0.03
End-diastolic diameter	4.7 (0.4)	4.6 (0.5)	0.13
End-systolic diameter	2.9 (0.3)	2.9 (0.5)	0.85
End-diastolic volume, 4CH	87.6 (20.6)	89.2 (27)	0.69
End-systolic volume, 4CH	38.8 (10.6)	40.9 (18)	0.43
LVEF (Teicholtz)	67.6 (7.5)	66.2 (8.7)	0.34
LVEF (Biplane Simpson)	55.2 (6.1)	52.9 (7.8)	0.07
TAPSE (mm)	23 (2)	22 (4)	0.006
MAPSE (mm)	13 (2)	12 (2)	0.004
E value	0.70 (0.62, 0.79)	0.65 (0.55, 0.78)	0.28
A value	0.80 (0.67, 0.90)	0.90 (0.75, 1.0)	0.04
LAVI (mL/m^2^)	42.7 (31.8, 49.8)	44.7 (36.1, 53)	0.19
Dt (m/s)	245 (39)	239 (46)	0.39
LV twist	20.8 (7.7)	19.4 (6.2)	0.22
GLS	− 18.9 (2.1)	− 17.8 (2.7)	0.008

Continuous variables are presented as mean and standard deviation (SD)

LV, Left ventricle; 4CH, Four-chamber; LVEF, Left ventricle ejection fraction; TAPSE, Tricuspid annular plane systolic excursion; MAPSE, Mitral annular plane systolic excursion; LAVI, Left atrial volume index; Dt, Deceleration time; GLS, Global longitudinal strain

**Fig. 1:**
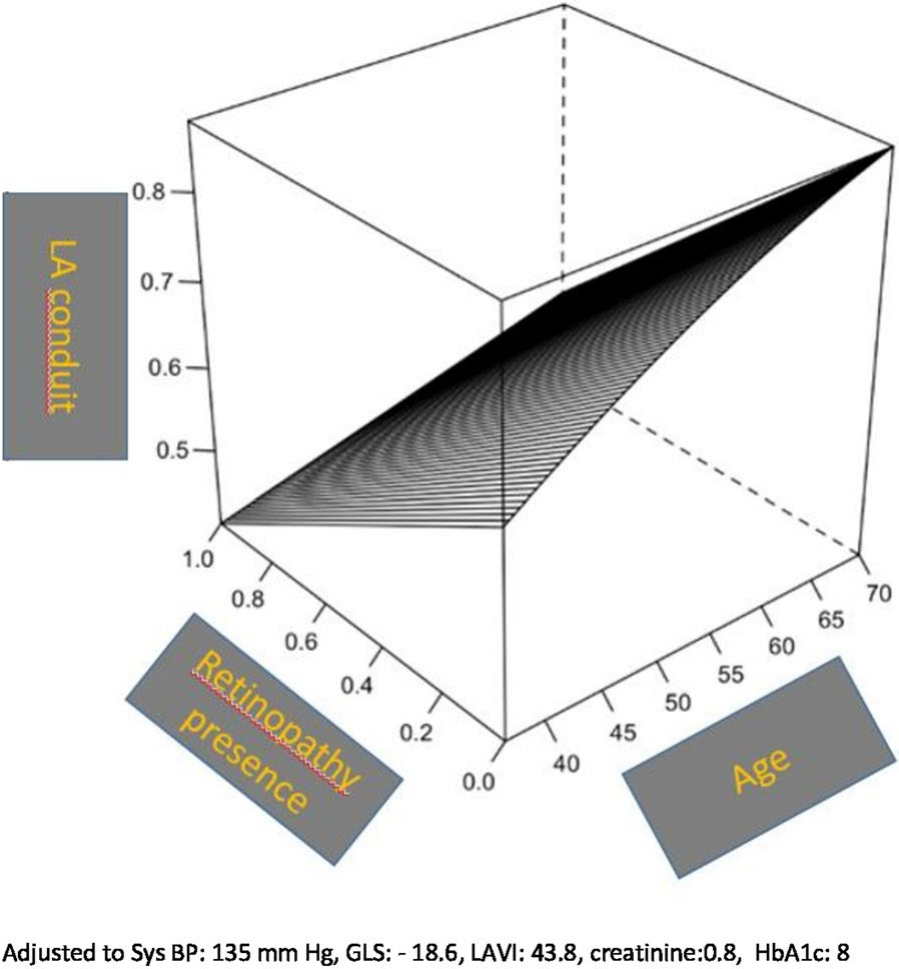
Three-dimensional plot for predicting LA conduit function according to DR presence and age

LA deformation parameters were analyzed in terms of LA reservoir, conduit, and contractile functions according to the degree of DR. The LA reservoir strain value was 14.2 ± 3.6 in normal retina group, 12.2 ± 4.1 in NPDR group, and 13 ± 3.7 in PDR group (*P* = 0.04). LA contractile strain was 15.9 ± 6.8 in normal retina group, 13.1 ± 47.4 in NPDR group, and 9.9 ± 4.7 in PDR group (*P* < 0.001). LA conduit strain was 30.1 ± 6.6 in normal retina group, 25.3 ± 6.5 in NPDR group, and 22.9 ± 4.9 in PDR group (*P* < 0.001). Three-dimensional plot for predicting LA conduit function according to DR presence and age is shown in Fig. 1.

**Fig. 2:**
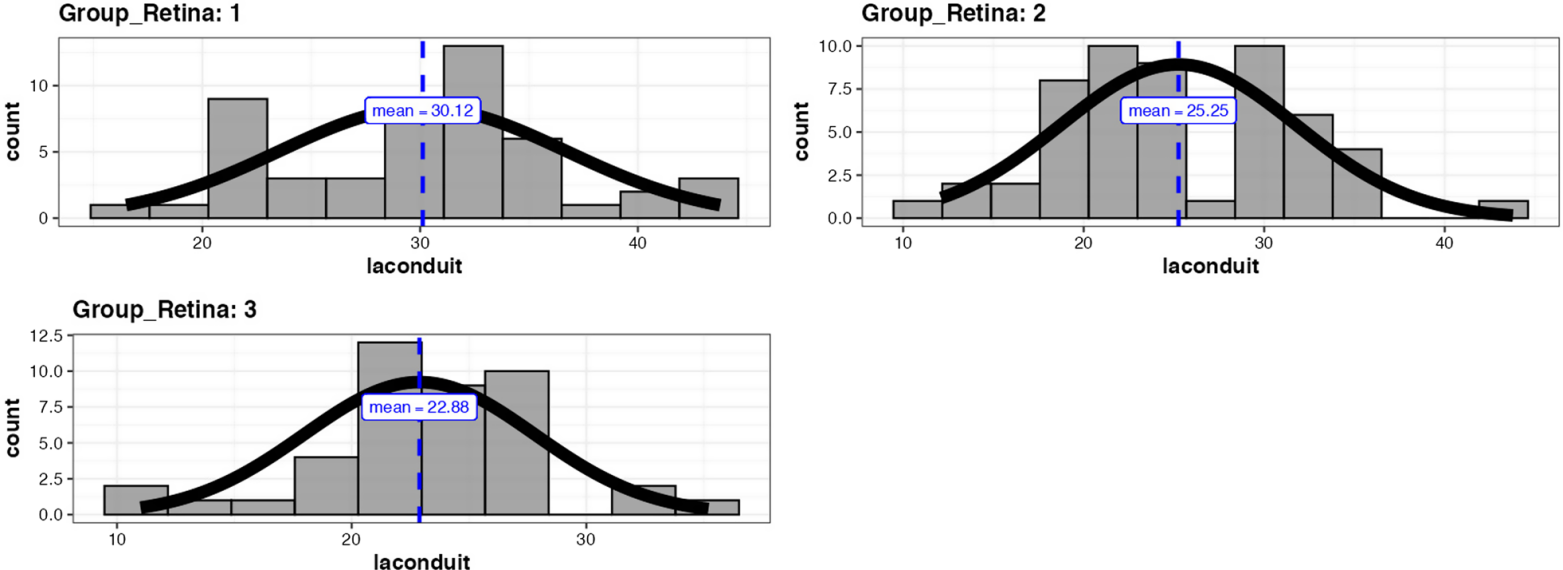
Density histogram of LA contractile strain values of the three groups

Density histogram of LA contractile strain values of the three groups is shown in Fig. 2.

The results of proportional odds regression analysis for association between clinical data, echocardiographic parameters, and LA contractile strain function demonstrated that increasing creatinine (from 0.7 to 1.0; OR 0.71; 95% CI 0.51–0.99; *P* = 0.04), retinopathy presence (OR 0.24; 95% CI 0.11–0.50; *P* = 0.001), and increasing LAVI (from 33.5 to 52.6; OR 0.62; 95% CI 0.43–0.89; *P* = 0.01) were associated with decreasing LA function; however, other variables did not show any association (Table 3).

**Table 3:** Comparison of advanced echocardiographic parameters in diabetics with normal retina versus non-proliferative and proliferative retinopathy

Variables	Normal retina, (*n* = 51)	Non-proliferative retinopathy present, (*n* = 54)	Proliferative retinopathy present, (*n* = 42)	*P*-value
LAVI (mL/m^2^)	42.6 (14.1)	46 (13.3)	53 (25)	0.006
E Mitral	0.7 (0.1)	0.7 (0.2)	0.7 (0.2)	0.07
A Mitral	0.8 (0.2)	0.9 (0.2)	1 (0.2)	0.10
LA strain rate S	1.5 (0.4)	1.2 (0.3)	1.1 (0.3)	0.01
LA strain rate E	− 1.2 (0.6)	− 0.9 (0.5)	− 0.9 (0.5)	0.01
LA strain rate A	− 1.7 (0.6)	− 1.5 (0.6)	− 1.7 (0.6)	0.19
LA strain conduit	30.1 (6.6)	25.3 (6.5)	22.9 (4.9)	< 0.001
LA strain reservoir	14.2 (3.6)	12.2 (4.1)	13 (3.7)	0.04
LA strain contractile	15.9 (6.8)	13.1 (7.4)	9.9 (4.7)	< 0.001

Continuous variables are presented as mean and standard deviation (SD)

LAVI, Left atrial volume index; LA, Left atrium

## Discussion

In this study, we determined an association between DR and LA contractile functions (OR 0.24; 95% CI 0.11–0.50; *P* = 0.001). Additionally, an increase in the degree of DR was correlated with a decrease in LA contractile, conduit, and reservoir functions (Table 4).

**Table 4:** Proportional odds regression model for predicting LA contractile strain function

Variables	Odds ratio	Confidence interval	*P*-value
Age (from 44 to 75 years)	2.07	0.72–5.89	0.17
HbA1c (%) (7.2 to 9.4)	1.09	0.72–1.64	0.66
Systolic BP (mmHg) (125 to 150)	0.90	0.61–1.31	0.58
Creatinine (mg/dL) (0.7 to 1)	0.71	0.51–0.99	0.04
Retinopathy presence	0.24	0.11–0.50	0.001
GLS (− 19.9 to − 16.6)	0.79	0.53–1.18	0.26
LAVI (mL/m^2^) (33.5 to 52.6)	0.62	0.43–0.89	0.01

BP, Blood pressure; LAVI, Left atrial volume index; GLS, Global longitudinal strain

The key role of LA remodeling in patients with DCM has been shown. Structural, mechanical, and functional changes develop in LA. Studies also revealed that LA function is a predictor for cardiovascular diseases in DM, even in patients without any cardiovascular risk factors [11, 12].

2D-STE is more sensitive than Doppler-derived strain measures because it allows angle independent direct analysis of myocardial deformation. The sensitivity of 2D-STE for LA mechanics has been recently validated. Abnormalities in LA strain have been shown in some clinical conditions, including LV hypertrophy, congenital heart diseases, dilated cardiomyopathy, and prolonged strenuous exercise [13–15].

The etiology for LA remodeling in DM is not well-known. Chronic hyperglycemia induces fibrosis in both ventricular and the atrial myocardium.

Hyperglycemia is also related with enhanced angiotensin II, TGF-β signaling, and increased reactive-oxygen species (ROC) production that probably promote atrial fibrosis in DCM, and it is associated with increased collagen synthesis.

Hyperglycemia can also be responsible for increased levels of advanced glycation end-products, which also provokes an increase in laminin and collagen, and thereby fibrosis. The decrease in LA elasticity related with fibrosis is a principal factor for all LA functions [16, 17].

The presence of DR suggests clinically manifested microvascular involvement in DM, and it is associated with impaired LV diastolic and/or systolic functions and might be a predictor of major adverse cardiac events. 2D-STE can detect subclinical functional changes in LA before the occurrence of structural changes [18, 19].

Georgievska-Ismail et al*.* evaluated the role of 2D-STE in LA systolic dysfunction in DM, suggesting that LA deformation mechanics are impaired in patients with DM and heart failure with preserved ejection fraction (HFpEF) [20]. Also, Y. Mochizuki et al*.* demonstrated both LA systolic and diastolic dysfunctions in type 2 DM with diabetic nephropathy and albuminuria [21].

In addition to these studies, in our study, the presence of DR (as a microvascular involvement) was found to be associated with a decrease in LA contractile functions (normal retina group: 15.9 (6.8); NPDR group: 13.1 (7.4); and PDR group: 9.9 (4.7); *P* < 0.001).

In patients with DM, preventing the occurrence of DCM is an important strategy, which might be achieved by preventing DR because the association with microvascular involvement for both clinical conditions is well-defined. After the development of DR, modification of cardiovascular risk factors and close cardiac evaluation might be helpful.

### Study limitations

Due to nature of regression model we might not include some important confounder, also this study is a single center study. We used retrospective design, even if consecutively admitted patients were included.

## Conclusions

Our results showed the relationship between left atrial deformation parameters and diabetic retinopathy, although microvascular involvement is not a certainly defined cardiovascular risk factor. Further prospective studies are needed to determine the clinical importance of DR presence and its degree for deformation parameters.

## Data Availability

None.

## Abbreviations

DM: Diabetes mellitus
LVSD: Left ventricular systolic dysfunction
HT: Hypertension
DCM: Diabetic cardiomyopathy
DR: Diabetic retinopathy
HF: Heart Failure
TTE: Transthoracic echocardiography
2D-STE: Two-dimensional speckle tracking echocardiography
LVEF: Ejection fraction
LA: Left atrium
LV: Left ventricle
TAPSE: Tricuspid annular plane systolic excursion
MAPSE: Mitral annular plane systolic excursion
LAVI: Left atrial volume index
GLS: Global longitudinal strain
BP: Blood pressure
LDL: Low-density lipoprotein
TSH: Thyroid stimulating hormone
WBC: White blood cell
hs-CRP: High sensitive C-reactive protein

## References

[1] Tan Y, Zhang Z, Zheng C, Wintergerst KA, Keller BB, Cai L (2020). Mechanisms of diabetic cardiomyopathy and potential therapeutic strategies: preclinical and clinical evidence. Nat Rev Cardiol.

[2] Brownlee M (2005). The pathobiology of diabetic complications: a unifying mechanism. Diabetes.

[3] From AM, Scott CG, Chen HH (2010). The development of heart failure in patients with diabetes mellitus and pre-clinical diastolic dysfunction a population-based study. J Am Coll Cardiol.

[4] Hiramatsu K, Ohara N, Shigematsu S, Aizawa T, Ishihara F, Niwa A (1992). Left ventricular filling abnormalities in non-insulin-dependent diabetes mellitus and improvement by a short-term glycemic control. Am J Cardiol.

[5] Minciună IA, Hilda Orășan O, Minciună I (2021). Assessment of subclinical diabetic cardiomyopathy by speckle-tracking imaging. Eur J Clin Invest.

[6] Kebed KY, Addetia K, Lang RM (2019). Importance of the left atrium: more than a bystander?. Heart Fail Clin.

[7] Tadic M, Ilic S, Cuspidi C, Ivanovic B, Bukarica L, Kostic N, Marjanovic T, Kocijancic V (2015). Celic V Left and right atrial phasic function and deformation in untreated patients with prediabetes and type 2 diabetes mellitus. Int J Cardiovasc Imaging.

[8] Badano LP, Kolias TJ, Muraru D (2018). Standardization of left atrial, right ventricular, and right atrial deformation imaging using two-dimensional speckle tracking echocardiography: a consensus document of the EACVI/ASE/Industry Task Force to standardize deformation imaging. Eur Heart J Cardiovasc Imaging.

[9] Karagöz A, Bezgin T, Kutlutürk I (2015). Subclinical left ventricular systolic dysfunction in diabetic patients and its association with retinopathy: a 2D speckle tracking echocardiography study. Herz.

[10] Lang RM, Badano LP, Mor-Avi V (2015). Recommendations for cardiac chamber quantification by echocardiography in adults: an update from the American Society of echocardiography and the European association of cardiovascular imaging. J Am Soc Echocardiogr.

[11] Obokata M, Negishi K, Kurosawa K (2013). Incremental diagnostic value of la strain with leg lifts in heart failure with preserved ejection fraction. JACC Cardiovasc Imaging.

[12] Mondillo S, Cameli M, Caputo ML (2011). Early detection of left atrial strain abnormalities by speckle-tracking in hypertensive and diabetic patients with normal left atrial size. J Am Soc Echocardiogr.

[13] Winter R, Jussila R, Nowak J, Brodin LA (2007). Speckle tracking echocardiography is a sensitive tool for the detection of myocardial ischemia: a pilot study from the catheterization laboratory during percutaneous coronary intervention. J Am Soc Echocardiogr.

[14] D’Andrea A, Caso P, Romano S (2009). Association between left atrial myocardial function and exercise capacity in patients with either idiopathic or ischemic dilated cardiomyopathy: a two-dimensional speckle strain study. Int J Cardiol.

[15] Oxborough D, Whyte G, Wilson M (2010). A depression in left ventricular diastolic filling following prolonged strenuous exercise is associated with changes in left atrial mechanics. J Am Soc Echocardiogr.

[16] Ritchie RH (2020). Abel ED basic mechanisms of diabetic heart disease. Circ Res.

[17] Masuda T, Muto S, Fujisawa G (2012). Heart angiotensin II-induced cardiomyocyte hypertrophy suppresses coronary angiogenesis and progresses diabetic cardiomyopathy. Am J Physiol Heart Circ Physiol.

[18] Zhen Z, Chen Y, Shih K, Liu JH, Yuen M, Wong DS, Lam KS, Tse HF, Yiu KH (2015). Altered myocardial response in patients with diabetic retinopathy: an exercise echocardiography study. Cardiovasc Diabetol.

[19] Tanaka A, Ishii H, Tatami Y, Shibata Y, Osugi N, Ota T, Okumura S, Suzuki S, Inoue Y, Murohara T (2014). Impact of diabetic retinopathy on late cardiac events after percutaneous coronary intervention. J Cardiol.

[20] Georgievska-Ismail L, Zafirovska P, Hristovski Z (2016). Evaluation of the role of left atrial strain using two-dimensional speckle tracking echocardiography in patients with diabetes mellitus and heart failure with preserved left ventricular ejection fraction. Diabetes Vasc Dis Res.

[21] Mochizuki Y, Tanaka H, Matsumoto K (2016). Impaired mechanics of left ventriculo-atrial coupling in patients with diabetic nephropathy. Circ J.

